# Uberon, an integrative multi-species anatomy ontology

**DOI:** 10.1186/gb-2012-13-1-r5

**Published:** 2012-01-31

**Authors:** Christopher J Mungall, Carlo Torniai, Georgios V Gkoutos, Suzanna E Lewis, Melissa A Haendel

**Affiliations:** 1 Genomics Division, Lawrence Berkeley National Laboratory, 1 Cycltotron Road MS 64-121, Berkeley, CA 94720, USA; 2 Library and Department of Medical Informatics and Clinical Epidemiology Oregon Health and Science University, 3181 SW Sam Jackson Park Rd, Portland, OR 97239, USA; 3 Department of Genetics, University of Cambridge, Downing Street, Cambridge, CB2 3EH, UK

## Abstract

We present Uberon, an integrated cross-species ontology consisting of over 6,500 classes representing a variety of anatomical entities, organized according to traditional anatomical classification criteria. The ontology represents structures in a species-neutral way and includes extensive associations to existing species-centric anatomical ontologies, allowing integration of model organism and human data. Uberon provides a necessary bridge between anatomical structures in different taxa for cross-species inference. It uses novel methods for representing taxonomic variation, and has proved to be essential for translational phenotype analyses. Uberon is available at http://uberon.org

## Background

**Figure 1:**
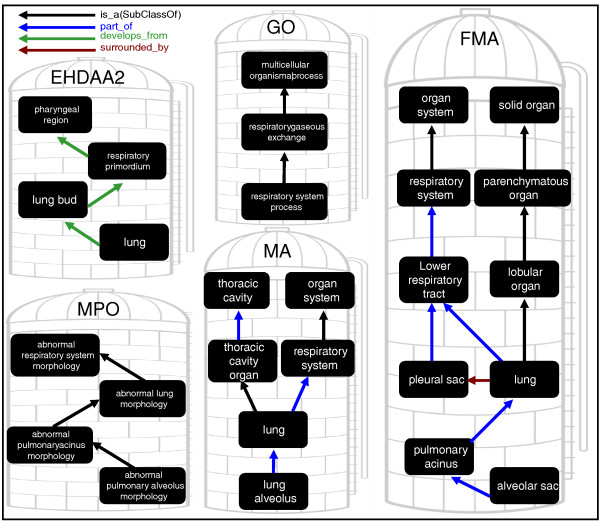
**Uberon integrates anatomical ontologies**. Anatomical representation of 'lung' and related types and processes are siloed in various ontologies with no connections. EHDAA/EHDAA2, Edinburgh Human Developmental Anatomy, abstract version/abstract version 2; FMA, Foundational Model of Anatomy; GO, Gene Ontology; MA, Mouse Anatomy Ontology; MPO, Mammalian Phenotype Ontology.

Anatomy ontologies (AOs) are computable representations of the parts of an organism and the structural and developmental relationships that hold between them. These representations have proven vital for databasing and bioinformatics analyses in fields including medical informatics, genomics, systems biology, neuroscience and comparative morphology [1]. The structural relationships encoded in AOs allow computers to determine that a query for 'all mouse genes expressed in the lung' should also return genes expressed in sub-structures such as the alveoli (Figure 1). AOs have proven useful for querying individual databases, but integrative queries spanning multiple databases or multiple species is problematic because each database uses a different ontology constructed according to different principles and requirements. There is a lack of inter-ontology connections between anatomy ontologies, and a lack of connections from anatomy to other domains such as phenotype. This results in a parcellation of data into isolated silos, as illustrated in Figure 1. Users wishing to query over multiple datasets will have to make multiple queries and integrate the results. For example, a query for mouse and human genes expressed in the lung at any stage of development or in abnormal tissues may require four or more queries in different places. Furthermore, without additional integration it is impossible to automate more sophisticated analyses, such as comparing all expression patterns of orthologous genes across species.

Table 1 summarizes some of the existing AOs, or ontologies that include an AO as a subset. Each of these ontologies has datasets that would benefit from integration. It may seem that the most effective approach would be for the community to standardize on a single anointed reference anatomy ontology, such as the Foundational Model of Anatomy (FMA) [2]. However, the FMA is designed primarily to represent post-embryonic human structures, and would be unsuitable for annotating zebrafish genes expressed in an embryonic fin bud. In order to serve the needs of their communities, model organism databases have developed dedicated species-centric anatomical ontologies (scAOs) such as the Zebrafish Anatomy Ontology (ZFA) [3], *Xenopus *Anatomy Ontology (XAO) [4] and the Fly Anatomy Ontology (FBbt) [5]. Each of these ontologies is designed to represent the anatomy of a particular species, and problems arise if we try and repurpose a scAO for other species, even closely related ones-for example, the FMA includes a relationship 'every mammary gland is part of some thoracic region', which is generally true for human mammary glands, but is clearly invalid for other mammals such as mouse. The Mouse Anatomy Ontology (MA) [6] has inguinal, cervical, thoracic and abdominal mammary glands represented as distinct classes. Both the FMA and the MA lack coverage of embryonic structures and developmental relationships, necessitating the use of other ontologies such as Edinburgh Human Developmental Anatomy (EHDAA2) [7] and the Edinburgh Mouse Atlas Project [8] ontologies for developmental studies in human and mouse, respectively. Further complicating this picture are dedicated ontologies that specialize in a particular anatomical system-for example, the Neuroscience Information Framework (NIF) Gross Anatomy, part of the NIF Standard suite of ontologies, represents neuroanatomy [9], integrating a range of brain atlases and databases. Other ontologies such as GALEN [10] and the National Cancer Institute Thesaurus [11] are not strictly anatomical ontologies, but include AOs as sub-ontologies.

**Table 1:** Summary of existing anatomical ontologies and comparison with Uberon

Ontology	Domain and applicability	Class count	Relations count	Relationship count	Text definitions	Computable definitions
Uberon	Animalia	6,546	IPD, 49	18,569	68%	*35%*
FMA	*Homo sapiens *(A)	80,467	IP, 15	124,392	1%	None
EHDAA2	*Homo sapiens *(AE)	2,397	IPD, 7	10,517	4%	None
MA	*Mus *(A)	2,982	IP, 2	3,775	None	None
EMAPA	*Mus *(E)	5,087	IP, 4	13,862	None	None
ZFA	*Danio rerio *(zebrafish) (AE)	2,656	IPD, 5	10,295	64%	None
TAO	Teleosti (bony fishes) (AE)	3,036	IPD, 5	4,828	49%	None
XAO	*Xenopus *(frog) (AE)	1,014	IPD, 6	2,238	72%	None
AAO	Amphibia (A)	1,601	IPD, 11	2,673	60%	None
FBbt	*Drosophila *(fruitfly) (AE)	7,110	IPD, 23	15,676	44%	24%
WBbt	*C. elegans *(nematode) (AE)	6,712	IPD, 6	12,187	70%	None
NCIt	Cancer-primarily Mammalia (AE)	3,506	IP, 3	5,913	67%	Yes
NIF [14]	Neuroscience-primarily Mammalia (A)	1,608	IP, 6	2,420	38%	Yes
BTO	All (AE)	630	IPD, 4	885	85%	None
EFO	Experimental factors all (AE)	1,004	IP, 5	1,127	55%	None
MESH	Indexing all (AE)	1,426	I	1,795	84%	None
BILA	Bilateria (AE)	114	IPD	132	44%	None
CARO	Metazoa (AE)	50	IP	49	100%	None^a^
PO	Viridiplantae (plant) (AE)	1,329	IPD, 7	2,180	100%	None
CL	Cells all (A)	1,925	IPD, 17	5,082	80%	48%

The first column states the ontology (full names and descriptions of these ontologies are given in the text). The second column states the domain: A, adult/post-embryonic structures; E, embryonic/developing structures. The third column shows the number of classes. The fourth column shows which of the three core relations are used (I, is_a/subclass; P, part_of; D, develops_ from) together with the number of relations used. The fifth column shows the number of logical relationships in the ontology. The sixth and seventh columns show the percentage of the ontology that has definitions (textual and computable, respectively). In cases where the scope of an ontology extends beyond anatomy, we list only the anatomical subset. ^a^The beta OWL version of CARO includes computable definitions. AAO, Amphibian Anatomy Ontology; BILA, Bilaterian Ontology; BTO, Brenda Tissue Ontology; CARO, Common Anatomy Reference Ontology; CL, Cell Type Ontology; EFO, Experimental Factor Ontology; EHDAA/EHDAA2, Edinburgh Human Developmental Anatomy, abstract version/abstract version 2; EMAP/EMAPA, Edinburgh Mouse Atlas Project, EMAPA is the abstraction from all stages; FBbt, FlyBase Anatomy Ontology; FMA, Foundational Model of Anatomy; MA, Mouse Anatomy Ontology; MESH, Medical Subject Headings; NCIt, National Cancer Institute thesurus; NIF, Neuroscience Information Framework; PO, Plant structure Ontology; TAO, Teleost Anatomy Ontology; WBbt, Worm Anatomy Ontology; XAO, *Xenopus *Anatomy Ontology; ZFA, Zebrafish Anatomy Ontology.

This pluralistic approach provides for coverage of a diverse section of biology, yet causes problems for data integration. These problems have the potential to worsen with the growth of high-throughput phenomic and next-generation sequencing projects, particularly in model organisms, and the need to integrate these data is more imperative than ever. One common approach to this problem has been to use entity matching and other automated methods to construct pair-wise mappings between the classes in different ontologies, but this approach is problematic for a number of reasons [12-14]. The mappings are error prone, lack semantics, and are difficult to maintain.

One alternative to automated pairwise mappings is to develop a comprehensive unifying shared AO, in which each class explicitly generalizes over classes in other ontologies, and is interconnected by means of logical relationships, allowing the use of automated techniques to integrate data. This ontology would leverage the specialized knowledge encoded in each of the existing ontologies, and would provide an additional integrative layer. Such an ontology would also form a vital building block in the modular development of a number of ontologies, such as Gene Ontology (GO), the Cell Ontology (CL) and the Ontology of Biomedical Investigations (OBI), each of which has a need to reference anatomical terms representing multiple species or taxa [15-17]. This ontology could also be used to seed new AOs for other key model organisms such as *Gallus gallus*, or could serve as a central source of anatomical structures for other less well-represented taxa, such as echinoderms and non-vertebrate chordates that may never have a dedicated scAO.

The first step in the construction of such an ontology was the Common Anatomy Reference Ontology (CARO) [18], which provides a set of high level abstract categories to serve as the standard upper level for all anatomy ontologies. More recently, this has been complemented by ontologies developed by the evolutionary biology community, such as the Teleost Anatomy Ontology (TAO) [19], the Amphibian Anatomy Ontology [20], which provide an integrative layer for particular vertebrate taxa. The Plant Structure Ontology (PO) [21] was originally developed to cover angiosperms, and is being generalized to be applicable across Viridiplantae. However, there has historically been a lack of comprehensive anatomical ontologies applicable across all animals, or even vertebrates. The closest has been the Brenda Tissue Ontology (BTO) [22], a terminology applicable across plants, fungi and animals, including gross anatomy, as well as cell types and diseases. Similarly, the Experimental Factor Ontology (EFO) represents species, developmental stage, disease and tissue type for the purposes of annotating gene expression data sets [23]. The EFO is used to represent data from 12 species and reuses or maps to existing AO classes to maximize interoperability. However, both the EFO and the BTO have a broader scope and have limited granularity with the domain of anatomy. Although the EFO is represented using the Ontology Web Language (OWL), it does not make use of the expressive features of this language that can be used in automated reasoning. In addition, the BTO does not integrate existing AO resources and has limited reasoning capability, and neither support extraction of taxon-specific information or addition of new taxon-relevant anatomical entities.

We created Uberon, the Uber-anatomy ontology, after identifying the need for a dedicated cross-species AO constructed on logical principles. The initial goal was to create a resource that could be used to connect biological datasets annotated with different ontologies. However, Uberon can be used independently as a standalone multi-species AO, and is being used as a source of classes and properties for ontologies covering other domains that have a need to reference generic anatomical types. In contrast to most mapping resources, Uberon is manually curated and we use automated reasoning as a means of quality control.

Uberon provides a sophisticated solution for many data integration endeavors. In this paper, we describe the contents of the ontology and the means by which it is integrated with multiple other ontologies. Rather than providing a single monolithic ontology, we provide different versions according to purpose. Here we first describe the main ontology, followed by extensions that incorporate additional ontologies. We then describe the principles and design decisions underlying the ontology, followed by a description of how Uberon is used in the modular construction of other ontologies. We then provide examples of how this ontology can be used for powerful cross-species queries and phenotypic analyses.

## Results

### Main ontology

The main version of the ontology consists of over 6,500 classes [24] (all ontology statistics are based on the September 2011 release version and exclude classes that have been obsoleted or deprecated), representing a variety of anatomical structures, grouped according to high-level categories from CARO. These include anatomical systems such as 'nervous system' and 'circulatory system'; organs such as 'heart', 'eye', 'brain', 'mesonephros' and 'pancreas'; tissues such as 'adipose tissue', 'cardiac tissue' and 'mesenchymal tissue'; developmental structures such as 'neural tube', 'pancreatic bud' and 'embryonic cloaca'; appendages or organism subdivisions such as 'feather', 'pelvic girdle' and 'limb'. For structures that are distributed over or repeated in multiple body parts, we provide explicit pre-coordinated compositional classes-for example, 'epithelium of lung', 'colonic mucosa', 'femoral epiphysis', 'forelimb skeleton', and 'apical ectodermal ridge of hindlimb'. Each class is in the UBERON namespace, and is uniquely identified by a URI of the form: http://purl.obolibrary.org/obo/UBERON_nnnnnnn

In this paper we shorten URIs to ID form, and for readability we refer to classes using the class label (enclosed in single quotes), with relations in *italics*. In contrast to corresponding classes in scAOs, these classes are explicitly intended to be applicable across a range of taxa where appropriate. For example, the class 'lung' is applicable to both avian and mammalian lungs.

We provide multiple download and import options for the ontology, each varying in complexity and scope, ranging from a simple subset of the core ontology to a multi-ontology import. The download table is available as Additional file 1, and is also summarized on the main web page (http://uberon.org).

The ontology is richly axiomatized, using a variety of constructs from the language OWL2-DL. In this paper we describe these and present examples using OWL Manchester Syntax [25]. These axioms include (but are not limited to) the *is_a, part_of *and *develops_from *links typically found in AOs used to represent the composition and ontogeny of structures [26]. These are all represented in OWL as SubClassOf axioms together with existential restrictions (for example, 'pulmonary alveolus' SubClassOf*part_of *some 'lung', meaning every pulmonary alveolus is part of a lung-but *not *implying that all lungs have alveoli). We describe the other logical axioms in more detail in the sections that follow. The full set of relations is available as Additional file 2. In addition to these logical axioms, the ontology also includes non-logical annotations typically found in AOs, such as textual definitions, synonyms, comments and provenance metadata. Table 1 shows some of the characteristics of Uberon compared against existing anatomical ontologies. It is larger than some model organism ontologies such as MA (mouse) and ZFA (zebrafish), but is dwarfed by the more detailed FMA, with 80,000 classes. Over 70% of the classes in Uberon have textual definitions, and over one-third have computable definitions that can be used by reasoners for automated classification.

The main ontology is available in both Open Biomedical Ontologies (OBO) format and OWL [27]. We also provide a basic version of the ontology, which contains all the same classes, but only a simple subset of the relationships (currently *is_a, part_of *and *develops_from*) [28]. Both of these ontologies have been classified in advance using a reasoner. A number of optional extensions are provided, and are discussed in more detail below.

### Multi-species bridging extensions

Most classes in the ontology are applicable across multiple species, and many are generalizations of classes in individual scAOs. For example, both FMA and ZFA contain classes called 'pelvic girdle', but with definitions inapplicable outside tetrapods and teleosts, respectively. The Uberon class 'pelvic girdle' (UBERON:0001271) subsumes the FMA and ZFA classes, and includes a generalized definition that is derived from the FMA definition, but has been modified to be applicable across vertebrates.

**Figure 2:**
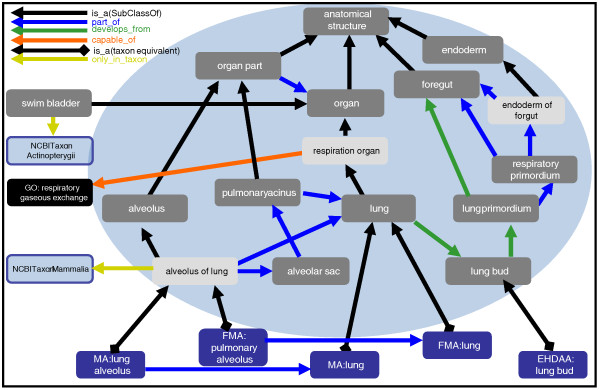
**Illustration of how Uberon relates anatomical silos into a unified view**. Uberon classes are shown in gray and classes from external ontologies are indicated with their respective prefix. Classes in light gray have computable definitions, which are indicated by the relations shown. For example, 'alveolus of lung' *is_a *'alveolus' that is *part_of *some 'lung'. 'Respiration organ' *is_a *organ that is *capable_of *GO:respiratory gaseous exchange. The blue circle indicates what would be included in a mammal-restricted subset of Uberon, as swim bladder is not found in mammals. Use of Uberon together with taxon-specific anatomy ontologies enables bridging of the data with full reasoning capabilities. In this example, Uberon 'lung' subsumes the lung classes from the mouse and human anatomy ontologies. Classes in the blue circle plus the blue classes at the bottom would be available in uberon-collected-mammal.owl. Note that some relationships have been trimmed for illustration purposes.

Figure 2 depicts the Uberon class 'lung' together with classes from individual scAOs, and the relationships connecting them. The resulting structure allows integrative queries over multiple databases annotated using different ontologies, one of the main use cases driving the development of Uberon. For example, a query for genes expressed in the Uberon (generic) 'lung' should return gene expression data annotated to the scAO lung classes, as well as individual parts, such as the mouse 'lung alveolus' (MA:0000420).

We have included over 17,000 connections between Uberon and scAO classes, derived through a combination of lexical matching, reasoning and manual curation (see Materials and methods). These connections are available in two different ways. In the main ontology, they are present as semantics-free cross-references ('xrefs' in OBO format). In addition, they are available as logical axioms distributed in separate bridging ontologies. These bridging axioms are imported together with the main ontology plus the relevant anatomical ontologies by means of taxonomically scoped 'collection' ontologies such as:

collected-metazoa.owl

collected-vertebrate.owl

collected-mammal.owl

**Figure 3:**
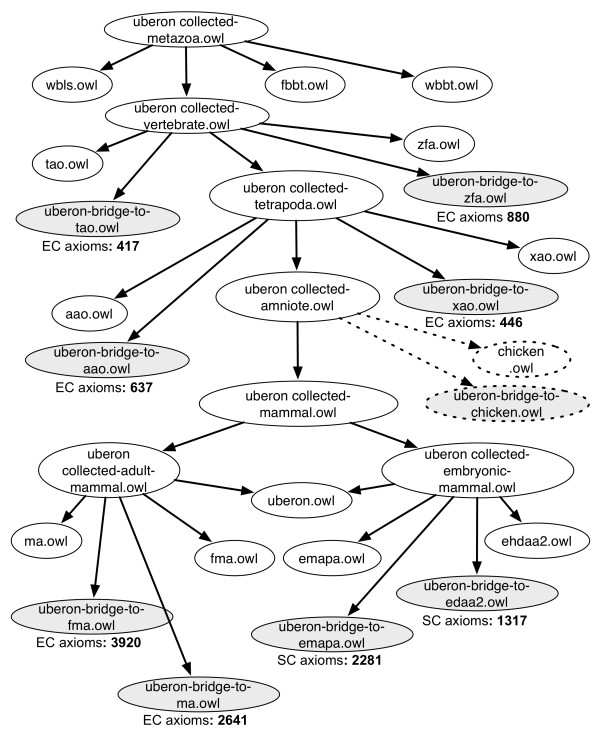
**Import chain of taxonomically arranged Uberon modules**. Each combined module at different taxonomic levels imports the relevant native ontologies as well as bridge files that specify the logical definitions. The number of equivalent class (EC) or SubClass (SC) axioms in each bridge file are shown, illustrating the contributions of each ontology to the total infrastructure. The files linked with dotted lines represent the mechanism by which a new chicken anatomy ontology (and similarly, archosaur) would be integrated.

The import hierarchy for each of these collection ontologies is illustrated in Figure 3. Each collector ontology imports the core ontology, bridging ontologies, and the individual species anatomy ontologies. The bridging ontologies contain either SubClassOf or EquivalentClasses axioms connecting the generic Uberon class to a taxonomic subtype or equivalent. For example, the mouse class 'lung' (MA:0000415) is declared equivalent to an Uberon class 'lung' (UBERON:0002048) that is *part_of *a mouse (NCBITaxon:10088).

As a general rule, we only include classes in Uberon where there is a need to generalize over existing ontology classes. Thus, 'lung' is present in Uberon as the super-class of the corresponding classes in MA, FMA, and Amphibian Anatomy Ontology (AAO). In some cases, there is the need to generalize a class from a single source ontology when it is relevant to mulitiple taxa-for example, 'brainstem nucleus' as found in the FMA. However, we do not include 'Weberian apparatus' because this structure is not found outside Otophysi, and this clade is already within the scope covered by the multi-species TAO. There would be no value in including this in the core ontology, as the class would be equivalent to the TAO class. Note that the combined vertebrate module that imports TAO would include the Weberian apparatus as part of the pan-vertebrate vertebral column.

One advantage to this taxon modularization approach is that it is relatively easy to include new AOs as they become available, and moreover, to seed them directly from existing applicable Uberon classes. For example, the currently in-preparation archosaur and chicken ontologies will be made interoperable with Uberon as per Figure 3. Bridging axioms will be created to these AOs, and a derived amniote ontology would include the union of the taxon-restricted amniote portion of Uberon, and the archosaur and chicken AOs.

### Multi-species composite ontologies

The combined modules above allow for reasoning and queries involving classes from multiple ontologies, but the resulting ontology structure can pose problems for ontology search and navigation, due to the presence of multiple named classes for each taxonomic variant of a structure. For example, when *collected-vertebrate *is loaded into an ontology visualization environment, the midbrain is visible at least four times, once each for mouse, human and zebrafish, and once for the generic vertebrate layer. The parts of the midbrain are also represented using a different class in each species, resulting in an ontology structure that is difficult to navigate because of the duplicity of labels and a complex lattice of multiple inheritance. In addition, query efficiency and reasoner classification time may be adversely affected by the prolifieration of classes. To avoid these problems we provide 'composite' ontologies, in which the taxonomic equivalents are automatically merged into the generic Uberon class. If a class has no taxonomic equivalent in Uberon, we do not merge it, placing it at the appropriate place in the ontology. For example, in the composite-vertebrate anatomy file, the multiple scAO classes for 'midbrain' have been merged into a single Uberon class, representing the pan-vertebrate structure. This ontology also includes classes not in the Uberon namespace, such as 'torus longitudinalis', which is represented by the zebrafish anatomy class ZFA:0001360 and is linked to the generic 'midbrain' class via *part_of *relationships.

Each model organism anatomy contains relationships that cannot be guaranteed to apply outside that taxon. For example, the XAO includes an axiom that the parathyroid develops from the '3^rd ^pharyngeal arch' (called '1^st ^branchial arch' in XAO)-but this cannot be generalized to all species with a parathyroid (Uberon includes a weaker axiom that states that all parathyroids develop from some pharyngeal arch, where the particular arch is not specified). When we merge the species class into the generic class we render these axioms safe by translating them into OWL General Class Inclusion (GCI) axioms. The *composite vertebrate *ontology contains the following axiom:

'parathyroid gland'(UBERON:0001132) and *part_of *some '*Xenopus*'(NCBITaxon:8353) SubClassOf*develops_from *some 'pharyngeal arch 3'(UBERON:0003114)

That is, every parathyroid found in an instance of *Xenopus *developed from a third arch. This does not imply that a human parathyroid develops from the same arch.

### Spatial and topological relationships

The ontology includes a rich set of spatial relationships-for example, every 'cranial nerve II' is *continuous_with *some 'retina'; every 'nerve fiber layer of the retina' is *adjacent_to *some 'inner limiting layer of the retina'. These can be used to enhance gene expression or phenotype queries, allowing the user to expand the query to include overlapping, continuous or adjacent regions. As well as being useful for end-user queries, many of these relations are vital for defining classes-for example, the interdigital regions between digits in human and mouse are defined by which digits they are adjacent to (see example in Table 2). We also include a subset of the relations defined in the spatial ontology (BSPO), such as *anterior_to*. Of these spatial relations, the most widely used are *in_left_side_of *and *in_right_side_of*, which are used to define the lateral halves of bilaterally symmetric or paired structures. For example, the left lobe of the thyroid gland is defined as a 'lobe of thyroid gland' that is *in_left_side_of *some 'thyroid gland'. The class 'left kidney' is defined as a kidney that is *part_of *some 'left side of organism', which is itself defined using the *in_left_side_of *relation. A full list of all relations is provided in Additional file 2.

**Table 2:** Example axioms

Class	OWL axiom	Module/ontology
pupil	SubClassOf: *part_of *some eye	Basic
'proximal phalanx of hand digit 1'	'EquivalentTo: 'proximal phalanx' and *part_of *some 'hand digit 1'	Basic
'left lung lobe'	SubClassOf: 'lobe of lung'	Basic
'left lung lobe'	EquivalentTo: 'lobe of lung' and *in_left_side_of *some lung	Main
'respiratory organ'	EquivalentTo: 'organ' and *capable_of *some 'GO:respiratory gaseous exchange'	Basic
'dermal skeletal element'	EquivalentTo: 'skeletal element' and *develops_from *some 'dermal tissue'	Basic
GCI	(*part_of *some 'brain') DisjointWith: (*part_of *some 'spinal cord')	Main (OWL only)
'superior eyelid tarsus'	EquivalentTo: 'eyelid tarsus' and *part_of *some 'lower eyelid	Basic
'left eye'	EquivalentTo: 'eye' and *part_of *some 'left side of body'	Main
bone	SubClassOf: *in_taxon only *NCBItaxon:'Vertebrata'	Merged (OWL only)
Interdigital region between forelimb digits 2 and 3 of 5	EquivalentTo: 'interdigital region' and adjacent_to some 'forelimb digit 2/5' and *adjacent_to *some 'forelimb digit 3/5'	Main
'thoracic mammary gland'	EquivalentTo: 'mammary gland' and *part_of *some 'thorax'	Basic
FMA:'mammary gland'	EquivalentTo 'thoracic mammary gland' and *part_of *some NCBItaxon:'Homo sapiens'	FMA bridge
GCI	(adenohypophysis and *part_of *some NCBItaxon:'Tetrapoda') deve*lops_from *some 'Rathke's pouch'	Merged
CL:'cerebellar granule cell'	EquivalentTo: CL:'granule cell' and *part_of *some 'cerebellum'	CL
GO:'immune response in Peyers patch'	EquivalentTo: GO:'immune response' and *occurs_in *some 'Peyers patch'	GO logical definitions

Classes are written as quote-enclosed labels for illustrative purposes. All classes are from Uberon, unless indicated by prefixing the label with the ontology name. The focal class is shown in the first column, and the axiom in the middle column. For General Class Inclusion axioms there is no focal class so we show the entire axiom in the middle column. We indicate the module/version in which the axiom appears-the simple module excludes most relationship types; the main module includes everything in simple, but no external ontology classes. The final two axioms are from external ontologies that reference Uberon.

### Life cycle stages

Uberon also includes a small sub-hierarchy of 29 life cycle stages (seeded from the stage ontology in the upper-level Bilaterian Ontology BILA), connected via *is_a, part_of *and *preceded_by *relations. Many of these stages are linked to and defined by a GO process (for example, the 'neurula stage' is linked to the GO process 'neurulation' via the *coincides_with *relation). There are relationships between anatomical entities and stages (for example, 'extra-embryonic structure' starts and ends during 'embryo stage'. Uberon stages subsume those of scAOs-for example, Uberon:'larva stage' would subsume the zebrafish stages 'larval:protruding mouth (72 hrs-96 hrs)' through 'larval:days 21-29'. Many temporal relations are required for all possible combinations of connections between stages, processes and anatomical entities; these are in the process of being formally defined (F Neuhaus, A Ruttenberg, and D Osumi-Sutherland, personal communication). See Additional file 2 for a description of these relations. Note that these links between anatomical structures, stages, and biological processes are not fully implemented and are intended as a first step towards temporal reasoning across developmental structures. At this time, these relations are course-grained, that is, we do not attempt to subsume individual Thelier and Carnegie stages [29].

### Inter-ontology relationships

We have included relationships and other logical axioms that reference other ontologies in Uberon, such as the GO, the Neuro Behavior Ontology (NBO) [30], the CL [15], the Protein Ontology [31] and CHEBI [32].

For connections between anatomical structures and GO or Neuro Behavior Ontology, we use the *capable_of *relationship and the *has_function_in *relationships [33], such as, for example, 'parathyroid gland' *capable_of *'parathyroid hormone secretion'. For connecting to CL, we use *has_part *to indicate the cellular composition of different organ parts and tissues. In the future we may use a more specific relation such as *has_granular_part*.

Note that all inter-ontology relationships are excluded from the main ontology, but are included in a merged ontology that also includes subsets of the external ontologies referenced together with the graph closure of all referenced classes. The merged ontology is available at [34].

One of the uses of the merged ontology is enhancing similarity-based queries and link-mining analyses. Without the use of these inter-ontology axioms, a gene that is implicated in 'ataxia' would show little ontological similarity with a gene implicated in 'abnormal cerebellar morphology'-but if there is a link between the cerebellum and the behavior 'gait', then a path can be established between these two phenotypes.

### Managing taxonomic variation

One of the main challenges involved in developing any multi-species ontology (and, in many cases, single-species ontologies) is accommodating organism variation. In a 'canonical' human anatomy ontology we can assert axioms such as ('mammary gland' SubClassOf*part_of *some 'female thoracic region'), but this is false for many non-human mammalian mammary glands (and, in rare cases, some human mammary glands). We accommodate this variation by making the generic 'mammary gland' class location-neutral, and then introducing subclasses for each location in which this gland can appear-for example, 'thoracic mammary gland', 'abdominal mammary gland', and so on. Note that we assign the FMA class 'lactiferous gland' as the taxonomical equivalent of 'thoracic mammary gland', rather than the more general 'mammary gland', because most human mammary glands are part of the thoracic region. We call this the named subclass approach to variation.

In some cases this scheme can lead to inflation in the number of ontology classes, leading to unwieldy multiple inheritance. For example, the adenohypophysis has different developmental origins in different species-while in most basal fish and tetrapods the adenohypophyseal anlagen invaginates to form Rathke's pouch, in teleost fish the adenohypophyseal placode does not invaginate but rather maintains its initial organization, forming a solid structure in the head [35]. If we were to use the named subclass scheme, we would introduce a class 'Rathkes pouch-derived adenohypophysis', but if we were to do this for all developmental variation, the results would be awkward and unnatural for end-users. Instead we take a different approach and create an OWL GCI axiom:

('adenohypophysis'(UBERON:0002196) and *part_of *some 'Tetrapoda'(NCBITaxon:32523) SubClassOf*develops_from *some 'Rathkes pouch' (UBERON:0006377)

The GCI approach accommodates taxonomic variation without inflating the ontology, at the expense of requiring OWL-aware tools to properly interpret the ontology. Note that these are similar to the GCIs that are created automatically when making the composite multi-species ontologies (see preceding section). The difference is that these are created manually, and encompass a wider variety of taxa. Generalizing developmental relationships across taxa can be controversial-there may be exceptions to the above rule within tetrapods, in which case we would replace 'Tetrapoda' with the appropriate taxon or set of taxa.

### Automation of ontology maintenance via logical axioms

In addition to simple relationships connecting classes, we have enhanced the ontology with a wide range of additional logical axioms. These primarily fall into three categories, examples of which are shown in Table 2: computable definitions, disjointness axioms and taxonomic constraints.

These axioms are intended primarily to assist with automated maintenance, quality control and classification of the ontology. This is particularly important for Uberon, which must remain in sync and consistent with multiple other ontologies.

#### Computable definitions

Over one third of the classes in Uberon have computable definitions-encoded as equivalence axioms between a named class and an intersection of two or more class expressions. These definitions allow a reasoner to automatically compute subsumption relationships between classes-for example, 'epiphysis of finger' can be automatically classified as a subtype of 'epiphysis of digit'. Asserting these manually would take considerable curator resources, and would be error-prone. The use of computable definitions in Uberon aids maintenance and can reveal potentially missing classes in the scAOs.

#### Disjointness axioms

If two classes are declared disjoint, it means that nothing can be an instance of both. If a class is inferred to be a subclass of two disjoint classes, the reasoner will flag it as unsatisfiable-this is a useful tool for detecting mistakes in the ontology, particularly in the context of an ontology that attempts to unify multiple other ontologies. We have created 410 disjointness constraints between classes in the ontology. In addition, we have created 751 spatial disjointness axioms in the ontology. For example, the brain and the spinal cord share no parts, or the central and peripheral nervous systems share no parts-though there may be some structures that overlap both, such as axon tracts. Uberon uses a standard merological definition of parthood, such that if A is *part_of *B, then every part of A is *part_of *B. If A *overlaps *B, then A and B share some part in common. Many of these axioms in the neural portion of Uberon were derived from the Allen Brain Atlas [36], and have proved useful in fixing problems with the ontology and individual species ontologies.

#### Taxonomic constraints

**Figure 4:**
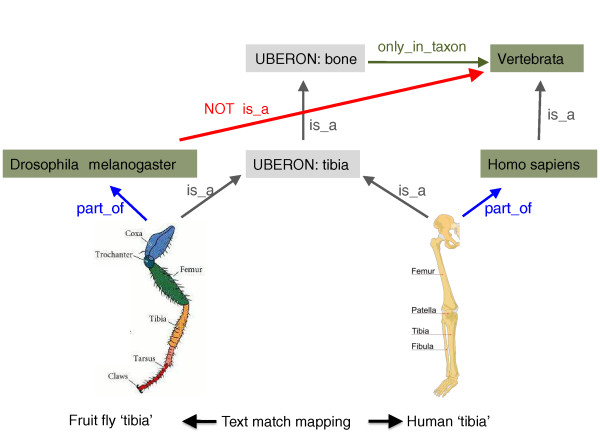
**Strategy for applying taxonomic constraints**. If the fruitfly class FBbt:tibia (representing a segment of an insect leg) were accidentally placed as a child of UBERON:0000979 'tibia', the reasoner would flag this as an error because 'tibia' *is_a *'bone' in Uberon, bones are found only in vertebrates, and FBbt:tibia is a *Drosophila *structure.

We have adopted the GO system of taxonomic constraints [37], and added 216 *only_in_taxon *and *never_in_taxon to *constraints to the ontology. These constraints are useful documentation for human users of the ontology, but their primary purpose is for automated consistency checking within the ontology and across ontologies. For example, if the FBbt class 'tibia' (FBbt:00004642), which represents a segment of an insect leg, were to be accidentally placed as a subclass or equivalent of 'tibia' (UBERON:0000979) based on the fact they share the same label, then a reasoner would infer that this class is formally unsatisfiable based on the three statements: (1) UBERON 'tibia' SubClassOf bone; (2) bones are never found in organisms that are not vertebrates; and (3) FBbt:00004642 can be found in *D. melanogaster *(Figure 4). In addition to automatic error-checking, these constraints can be used to create taxon-specific sub-modules of the entire ontology as described above (see Materials and methods). For example, if the scope of interest of a particular application is limited to Aves, then we can generate a sub-module that excludes structures such as fins, teeth and mammal-specific brain structures.

We provide some pre-generated taxon subsets as part of the release process, including a basic-amniote subset and a basic-aves subset.

### Maintenance of cross-ontology links

Uberon connects to multiple other ontologies, particularly other anatomical ontologies. Many of these ontologies are constantly evolving. We perform regular all-by-all lexical matching between all anatomical ontologies (see Materials and methods) to identify potential new connections. However, we never rely entirely on lexical matching-we use the output of lexical matching as suggestions that are manually vetted, sometimes after opening a dialog with the maintainers of the external ontology. The use of disjointness axioms and taxonomic constraints in the ontology also assists in detecting incorrect associations. The equivalence axioms are also used to automatically associate between species classes and generic classes.

### Provenance of metadata and relationships

Ontologies are constructed using information from multiple sources, including research articles, reviews, textbooks, encyclopedias, medical dictionaries and discussions with experts. It is important to track the provenance of all information collected in an ontology, and this is particularly important for an ontology such as Uberon, which as a matter of expedience frequently includes 'tertiary sources' such as Wikipedia, and other ontologies.

We attempt to include provenance identifiers for all definitions, synonyms and relationships. In each scenario, the item of provenance is an identifier that refers to an external source, such as a PubMed identifier or an ontology identifier. Multiple cross-references can be added to any piece of information. We include comprehensive Wikipedia cross-references, even where we have chosen to supplant the Wikipedia summary with our own definition. These can be used to build web pages that combine the structured ontology information from the ontology with the text from Wikipedia. Of the 4,692 definitions in Uberon, 2,293 have an association with a Wikipedia page. Two of the most frequently used resources are the Mammalian Phenotype Ontology (MPO; 379) and the GO (324)-both of these ontologies include a detailed implicit ontology of anatomical structures. There are 190 classes that take definitions from the FMA, but in many cases these are generalized to be applicable to non-humans. We are gradually refining definitions directly using the literature and expert review; at this time 100 definitions reference a Pubmed ID or a reference to a standard textbook, though many of the the definitions that cite an ontology term ID are indirectly citing a primary source.

We attempt to provide provenance for each synonym. For example, a synonym for the class 'cortex of kidney' (UBERON:0001125) is 'cortex renalis', which is marked as being used in Termina Anatomica and FMA:15581 (the Termina Anatomica synonyms are almost all derived from FMA). The FMA is the most commonly used source of external synonyms (4,133). In some cases, use of synonyms is contradictory-here we mark them as such and indicate the source of the synonym. An example of an inconsistent synonym is 'arm'-the MA (and Uberon) use this to mean the part of the forelimb that includes stylopod and zeugopod-in FMA it means just the stylopod region or the forelimb. The situation is analogous for 'leg'.

We also attempt to provide provenance on a per-relationship basis. This is particularly important for developmental relationships, which may not be straightforward to determine within a species and are even more difficult to generalize across species. In the future, we aim to provide evidence types as well as links to the source of the information, akin to GO annotations. Many of the relationships in Uberon have been sourced from other ontologies, but in most cases these have been checked to ensure they are applicable at the broader taxonomic level.

### Use of Uberon enhances queries in single organisms

One of the original motivations for the creation of the ontology was to integrate datasets from different species. More recently, we have found that the use of Uberon can enhance query capabilities within a single species. For example, neither the FMA nor the MA have developmental relationships, so we cannot query for all pharyngeal arch derived-structures using these ontologies alone. However, using either one of these ontologies in combination with Uberon and the appropriate bridging ontology, we can perform a description logic query to find all pharyngeal arch-derived structures (such as the human premaxilla and the mouse palatal shelf epithelium). See Additional files 3 and 4 for a full list of structures.

### An integrated anatomy ontology enables modular ontology construction

One of the main motivating factors for a multi-species anatomy ontology is the modular construction of other ontologies. For example, the environment ontology (ENVO) needs to include a number of organism-associated habitats, ranging from the gut of a termite to a human armpit. Similarly, GO and CL [15] classes such as 'blood vessel development', or 'cerebellar granule cell' are applicable across multiple species, and need to be defined in terms of generic anatomical classes rather than species-specific classes. These ontologies have traditionally included an implicit embedded anatomical ontology, but this leads to redundancy and is error-prone.

The GO classes can be made to explicitly refer to a generic anatomical type from Uberon to provide computable definitions (in a previous work we described the modular construction of the GO [38]). These include, for example, 'hepatic immune response', defined as being EquivalentTo 'immune response' and *occurs_in *some 'liver'. Conversely, GO is used to define structures in Uberon by the function they carry out-for example, 'parathyroid gland' *capable_of *'parathyroid hormone secretion'. We regularly use these logical definitions to perform automated reasoning to find missing links in the GO. When doing this reasoning, we occasionally find some inconsistency between different ontologies that would be expected to conform. For example, we discovered inconsistencies between the GO and various scAO*s *in the treatment of the term 'gut'. The GO was therefore restructured to use the term consistently with Uberon. On manually resolving these inconsistencies between the existing relations and the relations implied by the embedded definitions, one or both ontologies are improved. We have now provided 1,473 logical definitions for GO classes using Uberon. These are supplemented by additional logical definitions for clade-specific classes outside the scope of Uberon, for which we use ZFA, FBbt and Plant Structure Ontology.

Similarly, the CL is applicable across species and refers to generic gross anatomical types for many of its location-specific classes. For example, 'splenic red pulp macrophage' refers to the macrophages within the gross anatomical structure 'splenic red pulp'. These location-specific classes require an ontology of anatomical structures, such as Uberon, to construct computable definitions. This augmentation is underway, and CL is adding computable definitions using Uberon and other OBO ontologies [33] using the *capable_of *relationship and the *has_function_in *relationships [33]. Conversely, the CL is used in Uberon to indicate the composition of tissues and organs, primarily through the *has_part *relation. Note that the CL and the extended version of the GO both provide links to Uberon, but these are not redundant. The majority of links from the GO to Uberon are in the development hierarchy, whereas links in the reverse direction typically connect organs to the functions they perform.

## Discussion

### Enhancement of existing ontologies

We consider definitions to be of central importance in all ontologies; textual definitions allow human annotators to reliably disambiguate similar terms, and computable definitions allow the use of automated methods to assist in ontology construction and data integration [39]. Unfortunately, existing AOs exhibit considerable variability with respect to definitions. Some ontologies such as MA have neither textual nor computational definitions. Only 1% of the classes in the FMA have textual definitions, while many other model organism ontologies have good coverage with text definitions alone. In building Uberon, we have leveraged both text and computable definitions in the source ontologies. Therefore, Uberon provides classes that can be more precisely used for annotation in a cross-species context or to improve existing species-specific annotation procedures.

In constructing Uberon, we have revealed inconsistencies in related ontologies such as GO, MPO, CL and others. Since GO and CL are applicable to multiple species, they need to describe developmental and physiological processes in a species-neutral way. However, these ontologies have traditionally contained an implied anatomical hierarchy without logical definitions and with inconsistent textual definitions. These ontologies are problematic in their inconsistencies but are also a source of valuable anatomical definitions. For instance, the GO has numerous anatomically relevant developmental processes such as 'midbrain development,' which is defined as: 'The process whose specific outcome is the progression of the midbrain over time, from its formation to the mature structure. The midbrain is the middle division of the three primary divisions of the developing chordate brain or the corresponding part of the adult brain (in vertebrates, includes a ventral part containing the cerebral peduncles and a dorsal tectum containing the corpora quadrigemina and that surrounds the aqueduct of Sylvius connecting the third and fourth ventricles).'

We have leveraged these implied anatomical descriptions and relationships in the seeding of Uberon and consistency checking of these species-neutral ontologies versus Uberon (see Materials and methods). The Uberon approach to ontology alignment and integration has proved to be a valuable mechanism to systematically evaluate and improve these ontologies, and it is now possible to leverage reasoning to ensure interoperability and orthogonality across these disparate yet putatively orthogonal ontologies.

### Limits of pure text-mining approaches

A systematic comparison of Uberon with lexical text matching approaches is outside the scope of this paper. Such a comparison would be partly confounded by the differing goals of the two approaches-pairwise mappings establish horizontal connections between similar classes in different ontologies, whereas Uberon provides classes with definitions and relationships that connect vertically to other ontologies. Lexical mappings typically lack explicit semantics, and provide no way of separating closely related classes from equivalent classes. The need to augment purely lexical anatomy ontology mappings has been identified previously [13] and enhancement of lexical matching methods using semantics in various ways has been reported and continues to be investigated within the context of the annual Ontology Alignment Evaluation Initiative (OAEI) [40]. Whilst the initial version of Uberon was partly seeded by matching labels and synonyms, the value added by thoroughly verifying the results of this process semantically and biologically (manually) converts these exercises into practical tools. As there is an increasing amount of anatomically indexed expression and phenotype data, the need for analysis and query of such data will require increasingly specific semantics-where pure text-matching approaches will not suffice. A recent study by Groß *et al. *[41] has further validated the approach taken in the development of Uberon, where Uberon scored better as an intermediate source of ontology mappings on a number of metrics than other sources. This experiment demonstrated that 'Uberon finds non-trivial correspondences that cannot be identified by a direct match.'

### Homology and analogy

One possible critical perspective on Uberon is that its classes are essentialist-they are intended to group entities by common properties. It is thus possible that many of its classes are pleisomorphies. For example, Uberon contains grouping classes 'eye' and 'wing', despite the fact that neither of these are homophyletic-they evolved multiple times. The inclusion of a class in the ontology should not be taken as an indication of shared evolutionary descent (homology), merely that classes have some property or properties in common. We have taken an integrative approach in the building of Uberon, and in doing so embrace multiple axes of classification. Traditionally, anatomists have used many modes of classification, and these all appear within Uberon. These classification axes may be structural, functional, or developmental in addition to homology, to aid in grouping structures by similarity of any type. Using a single classification axis such as structure or homology alone is either too restrictive or may lead to incompleteness due to incomplete knowledge. For example, Uberon includes a generic functionally defined class 'eye', which is defined by its function 'detection of visible light'. This generic class subsumes the class called 'eye' in the *Drosophila *anatomy ontology and the class called 'eye' in the MA (note that it is not the direct subsumer, as these two classes are subsumed by the Uberon classes 'compound eye' and 'camera-type' eye, respectively). Assignments of biological function are made using the biological process subset of the GO.

The Uberon approach is complementary to resources such as Homolonto [42], which groups vertebrate species-centric AO classes into vertebrate Homologous Ontology Groups (vHOGs) based on shared evolutionary descent [43]. As described above, homology is only one means by which two anatomical structures can be deemed similar. We have sought to include many axes of similarity and therefore used the vHOGs in the seeding of our ontology. One difference between the two approaches is that Homolonto only seeks to assign classes into groups. Unlike Uberon, it does not attempt to define a common subsuming structure, nor is it intended for reasoning. Neither does Homolonto distinguish structures by ontogeny-thus, 'gonad' and 'gonad primordium' in different species are placed into the same group. This makes sense from the perspective of homology grouping-the structures are indeed homologous-but the distinction made in Uberon between structures and precursor structures allows for more precise queries across developmental time. Conversely, the vHOG approach provides certain homology groupings that are not present in Uberon-for example, between 'lung' and a fish 'swim bladder' (Figure 2). This reflects the complementary goals of the two projects and does not present a problem-in fact the two resources can be dynamically combined and the developers of Uberon and vHOG are collaborating to support this more extensive query capability.

**Figure 5:**
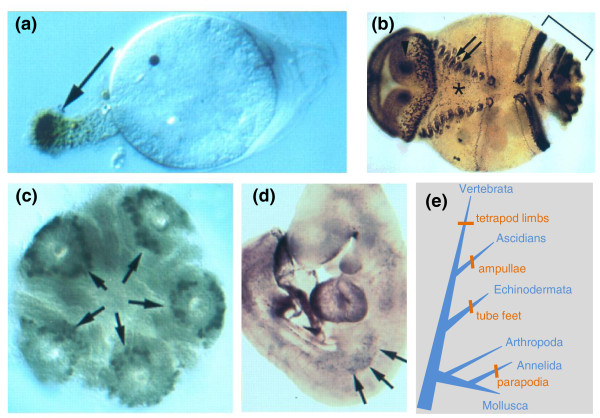
**Expression of Distal-less (Dll) and Dll orthologs (Dlx) in 'legs'**. **(a) **Three-day *Molgula occidentalis *ascidian larva from which an ampulla is extending. **(b) **Polychaete annelid *Chaetopterus variopedatus*, ventral view of larva just prior to metamorphosis (anterior to left). Dll expressing cells are visible in parapodial rudiments (arrows), antennae (out of focus on opposite dorsal surface), and in prospective feeding organs (bracket). **(c) **Metamorphosing *Strongylocentrotus droebachiensis *sea urchin larvae, aboral view. Cells at the distal tip of the tube feet (arrows) express Dll prior to and during extension from the body wall. **(d) **Expression in nine-day mouse embryo, lateral view, head top; arrows point to medial border of cells expressing one or more Dlx genes in the presumptive forelimb. **(e) **The evolutionary appearance of the various appendages for which Dll expression data are indicated in (a-d) are shown on this cladogram (branch lengths are not scaled). Reprinted with modification and permission from [49].

This homology-neutrality of Uberon is a deliberate design feature of the ontology. We believe that specifying homology relationships and descent from common ancestral structures is of obvious high value, but that this need not be tightly coupled to the development of an upper anatomical ontology. This does not preclude creation of subsuming classes based on homology (as in the Vertebrate Bridging Ontology project [44]), but rather that it is not a requirement and nor is the homology assertion definitional for any given class. One reason for this is that statements of homology can be controversial, subject to change and even contradictory. Uberon forms a neutral structure on which to pin evolutionary statements. Homology is of course very important from the perspective of navigating gene expression and phenotype data across species, but it provides only a limited set of potentially interesting results. In Uberon, the 'essentialist' definitions are biologically informative even without evidence of evolutionary relatedness. For example, it is useful to retrieve all eye phenotypes from multiple species regardless of evolutionary history. For example, the *Pax6 *master regulator gene is active in eye development in species as diverse as *Drosophila *and humans [45-47]. Similarly, the Dll gene orthologs are implicated in the development of tetrapod limbs, ascidian ampullae, annelid parapodia, and echinoderm tubefeet (Figure 5) [48]. These eyes and appendages are certainly not homologous, but they do have some functional similarity (which is in some cases why they have been given the same label historically). Why are these eyes and appendages similar? In some cases there may be homology of anatomical parts, biochemical pathways, or molecules that are at a level more granular than what has been studied using comparative anatomical phylogeny reconstruction (deep homology) [49]. For instance, photoreceptors may be homologous even if the eye structures themselves are not. Alternatively, convergent evolution may result in reuse of similar pathways for similar functionality. For example, outgrowth from the body wall as described in the 'limb' study by Panganiban above may be due to convergent evolution, or perhaps there is some yet to be defined homology in this process. For these reasons, we believe it is critical to be able to query across species via grouping of similar structures independent of what is currently known about homology.

### Uberon coordination and integration with other ontologies

Uberon is intended to work as part of a strategy of interlocking ontologies with various degrees of specificity and is applicable across all metazoans-that is, any organismal part of a metazoan organism is within scope for Uberon. In this initial release, Uberon does not claim to be comprehensive in its coverage of metazoans and there is a considerable bias towards vertebrates and especially mammals, due largely to bias in the ontologies subsumed by Uberon. However, Uberon is now being extended to cover a wider variety of species, such as birds and archosaurs, for representing evolutionary data. If and when such anatomical ontologies become available, Uberon will subsume them using the same methodology as for other scAOs.

In cases such as the NIF neuroscience ontologies [9], where domain experts are already constructing ontologies covering particular anatomical sub-domains, our practice is to provide cross-references to these resources. Similarly, the vertebrate musculoskeletal anatomy ontology developed as the outcome of a workshop at the National Evolutionary Synthesis Center [50] has been integrated into Uberon. As new groups of domain experts work to accurately represent their specialized area of anatomical knowledge in ontologies, we would likewise defer to these experts and gradually cede control to the relevant specialized ontologies. For example, there is a new effort to develop an arthropod anatomy ontology [51]. Uberon currently contains structures like 'ventral nerve cord' and 'arthropod sensillum' (synonym sensillum), which would be ceded to the arthropod ontology following an initial release. For 'sensillum', Uberon would retain the more generic parent class 'sense organ' since this has applicability outside the arthropods. Additionally, rather than our current practice of using cross-references to capture these links, we will import classes from domain-specific ontologies, such as the NIF neuroscience, skeletal, and arthropod ontologies, and use their IDs directly using the MIREOT approach to reference external ontology terms [52]. This is particularly relevant in the context of the upper CARO [18]. CARO is currently being revised to better represent community needs following implementation in various AOs over the past few years. In particular, it will now include inferred multiple inheritance, disjointness axioms, and functional differentia [53,54].

One advantage of Uberon is that it includes classes for which no dedicated AO already exists-for example, the parabronchial lungs of avians and a diverse range of structures from under-represented taxa, including ungulates, tunicates and echinoderms. This capability is especially useful for the representation of non-model organism data. For instance, the eagle-i project captures information about a diversity of non-model organisms [55,56] using Uberon classes whereby the relevant species is specified (for example, a muskox 'brain'). The utility of referencing a taxonomically general class in combination with a given taxon will be invaluable as new genomes are sequenced and non-model organism expression and phenotype data become available.

Conversely, developers of new scAOs can take advantage of the work that has been done by other scAOs and in Uberon. Much in the same way as CARO is used as an upper AO to structure a new scAO, the new scAO can also use Uberon classes to seed their scAOs. For instance, a new chicken ontology can MIREOT the taxonomically relevant portion of Uberon (for example, collected-vertebrate.owl) and then extend this ontology under its own namespace. An example of how this would work is that the chicken ontology could create a chick 'tertial feather' class that is subsumed by the Uberon 'feather' class (Figure 3). We would need to ensure that the definition of Uberon 'feather' is applicable to the chick 'tertial feather' class. If not, the Uberon class could be adjusted or a new term added to support the general type 'feather' and its subsumption of chick 'tertial feather'.

The OBI represents the entities involved in research, namely roles, functions, objectives, processes and the input and output of these processes [17]. OBI can be used to check consistency in experimental design, classify bio-specimens, and infer the relationship between assays and what is being evaluated. OBI necessarily needs to refer to anatomical structures in their 'planned process' branch-for example, 'blood harvesting' or 'bronchial alveolar lavage'. Similarly, the representation of bio-specimens involves reference to gross or cellular anatomy-for example, 'cloacal specimen'. These anatomical terms refer to taxonomically general types, not usually those of a single organism. Where applicable, OBI is now using Uberon classes for their definitions involving cross-taxon anatomy similar to the GO and CL.

Coordination with Uberon has resulted in improvements and clarifications in other ontologies. See Additional file 5 for a list of items from various ontology issue trackers pertaining to development of this ontology.

### Linking animal models to human diseases

A significant barrier in translational research is an inability to query between human, model and non-model organisms due to the difference in terminology used to describe their anatomy. In a previous study we described a methodology for enhancing and connecting animal phenotype ontologies [57]. We created computable definitions for existing phenotype ontologies such as the MPO, the Human Phenotype Ontology (HPO) [58-60] and the Worm Phenotype Ontology (WPO) [61]. These computable definitions referenced a range of ontologies, primarily the Phenotype and Trait Ontology (PATO) and individual scAOs such as MA, FMA and the Worm Anatomy Ontology (WBbt). In order to connect these phenotypes, we used an early version of the Uberon ontology. We then devised semantic similarity measures for the resulting phenotype descriptions that allowed us to link animal models with human diseases [62] based on phenotypes alone, and have further analyzed a wider range of human diseases [59,60]. We have recently leveraged Uberon for the purposes of querying across species and anatomical granularity in a neurodegenerative disease knowledgebase [63] Similarly, other projects requiring cross-species inference to investigate animal models of disease are beginning to use Uberon. Facebase [64] is a consortium that aims to consolidate and make queryable data regarding neural crest development and craniofacial diseases. The ontology is also being used by the FANTOM5 consortium [65] to indicate the tissue source of sequenced samples. To support the use of Uberon in such informatics applications, we provide scripts and code examples on http://uberon.org.

## Conclusions

Translation of knowledge across species is hindered by a lack of integration between anatomy ontologies. Uberon is a multi-species anatomy ontology that integrates different anatomical ontologies and the datasets annotated using these ontologies. The ontology has been integral in a number of computational analyses that interpret human data using model organism phenotypes for translational research. Uberon contains a rich set of logical relationships that allow powerful queries within and across species. Further, Uberon serves as a nexus for connecting multiple other biological ontologies such as the CL and the GO, and in the modular construction of other multi-species anatomy ontologies. We believe that Uberon meets the current need for an integrative cross-species anatomy ontology amongst the OBO Foundry suite [66].

## Materials and methods

### Ontology seeding via semi-automated methods

Because ontology construction is a labor-intensive task, we opted to automate as much as possible by drawing on expert knowledge already encoded in existing anatomy ontologies, as well as on the implicit anatomical ontologies embedded within phenotype ontologies and the GO. The initial version of Uberon was created, as a matter of expediency, using automated lexical methods. In contrast to most mapping approaches, however, we decided to work with and consult knowledgeable anatomical experts to curate and manually edit the results to come up with the best possible representation of established anatomical relationships. We also leveraged computational reasoning as much as possible, to automate various quality control checks and compositional term creation. These three methods, lexical matching, manual curation, and computational reasoning, are combined iteratively, although over the evolution of the ontology lexical methods have largely been superseded by manual curation and reasoning.

The initial phase of construction of Uberon broadly consisted of three components: initial seeding of potential Uberon classes from existing scAOs; augmentation from other external sources for both additional classes and for logical definitions; and manual revision by anatomists throughout the process.

Note that we used a broad spectrum of methods in an iterative fashion, altering the parameters and algorithms at each iteration, gradually enhancing the ontology as we progressed. Our goal here is not to describe generic reproducible lexical methods for generating multi-species ontologies.

### Ontology seeding via lexical matching

The initial version of the ontology was seeded by extracting generalized classes from sets of similar classes from existing scAOs. Using the Blipkit framework [67], we took the names and synonyms from each ontology, tokenized them, and performed Porter-stemming [68], removed certain tokens such as 'of' and 'the', and matched two classes if the set of stemmed tokens from any label was identical. We also mapped all relational adjectives to a standard noun form using a set of hand-constructed mappings (for example, 'facial' to 'face'). We created an Uberon class for every connected set of paired classes, maintaining the link back to the scAO as an OBO format 'xref'.

We then manually split classes in OBO-Edit [69], using anatomical knowledge guided by various rules of thumb and heuristics. For example, we paid particular attention to classes that unified vertebrate and invertebrate classes, as these were more likely to be wrong and solely due to homonymy. We also were careful to examine classes that were created based on non-exact synonyms. The OBO-Edit graph viewer was used to visually check the combined *is_a *hierarchy and partonomy were biologically correct.

This process was highly iterative, as we chose to align additional ontologies such as EHDAA2 after the initial seeding. We also re-did our matching after performing improvements to our text matching techniques; for example, adding per-token synonyms such as 'first' for '1^st^' enabled faster matching of hand and foot digits between mouse and human.

### Automatic seeding of computable definitions

Many AO classes are combinatorial due to serial homology; for example, bone regions such as the epiphysis and diaphysis appear multiple times in different digit segments, and digits themselves appear multiple times, on both fore and hind limbs, and on left and right sides of the body. Managing these compositional classes manually is time-consuming and error-prone. We set out to generate logical definitions for these classes in order to use automated reasoning to assist with ontology construction.

The majority of the logical definitions we generated were simple 'genus-differentia' style equivalence axioms between a named ontology class, and a class intersection between a 'genus' class and an existential restriction. For example, in OWL syntax:

Class: 'forelimb digit'

EquivalentTo: digit and part_of some forelimb.

We used a combination of manual assignment and automated generation of definitions, with the Obol toolkit [70].

We reverse engineered class definitions using simple Obol generative grammar rules such as:

P and *part_of *some W → W P

P and *part_of *some W → P 'of' W

(Note that the same rules can be used for generation as for parsing.)

This allowed us to derive computable definitions such as 'epithelium and *part_of *some lung' for the class with label 'lung epithelium'. These definitions are vetted for non-sensical parses, such as those generated from labels such as 'neck of uterus', which refers to an organ neck rather than the body subdivision between the head and the thorax. We generated these definitions for Uberon terms and used reasoning to automatically classify them.

We also generated these logical definitions for existing scAOs-note that most do not yet maintain their own logical definitions. The resulting logical definitions are available as bridge files in the uberon repository [71]. These are divided into two sets-those that do not reference classes outside the scAO (example in [72]), and those that reference a more generic uberon class (example in [73]).

We used these logical definitions in scAOs to seed new Uberon classes. We generated a new Uberon class for every scAO class whose definition elements map to Uberon classes. For example, we defined 'aorta endothelium' (MA:0000701) as (MA 'endothelium' and *part_of *some MA 'aorta'). We generated an Uberon class 'aorta endothelium', defined as UBERON 'endothelium' and *part_of *some UBERON 'aorta'.

### Augmentation using the Gene Ontology

The subset of the GO pertaining to developmental processes is a rich source of anatomical knowledge that is applicable across a wide range of species. We first generated a set of computable definitions for GO biological processes using Uberon (previously described in [38]). We used reasoning to suggest changes in the GO hierarchy, or conversely, to modify branches of Uberon such that asserted relationships in GO can be justified via inference.

We then augmented Uberon by taking the set of GO classes with labels following certain lexical patterns such as '*X *development' and '*X *morphogenesis'. If this GO class did not have a computable definition, and we did not have an Uberon class with label *X*, then we generated one, using reasoning to suggest the placement within the Uberon hierarchy, and extracted a textual definition from GO. The resulting classes were then used to create computable definitions for GO classes, which were used in reasoning to iteratively refine both GO and Uberon hierarchies.

We obtained textual definitions by extracting the embedded GO definition of the anatomical structure (which is usually, but not always, constructed to be species-neutral). For example, the GO definition for 'kidney morphogenesis' includes this after the main definition: '...A kidney is an organ that filters the blood and excretes the end products of body metabolism in the form of urine...'.

In general we excluded terms that fall in the domain of other AOs. For example, there is little value in adding *Drosophila*-centric terms into Uberon where these terms are not applicable across a wider range of scAOs.

### Augmentation using phenotype ontologies

Phenotype ontologies, like GO, also include an implicit embedded AO. We used the MPO [74] and the Human Phenotype Ontology [58] in a method analogous to the one described for GO, above. We searched for lexical patterns such as 'abnormal X morphology', and created a term 'X' if this did not already exist in an existing AO. We also extracted the text definition and a suggested hierarchy, in the same fashion as for GO.

### Augmentation using DBPedia

DBPedia is an RDF triplestore derived from Wikipedia [75] that translates Wikipedia infoboxes into RDF triples, and makes stable URIs for Wikipedia entries. We used the SWI-Prolog semweb library [76] to issue iterative SPARQL queries to extract all RDF triples for all instances of the DBPedia ontology class 'dbpedia:AnatomicalStructure'. We then mapped the resulting triples into an OBO format ontology, and aligned this in a similar fashion to the other anatomical ontologies. The DBPedia 'abstract' property was mapped to a definition field, redirects properties were mapped to synonyms, and the 'precursor' property was mapped to *develops_from*. We then used our text mapping algorithms described above to link as many Uberon classes as possible to Wikipedia pages.

### Augmentation with additional logical axioms

We automatically populated many taxonomic constraints using the taxonomic constraints already encoded in GO [37]. We inferred that if a GO biological process is restricted to a particular taxon, then the anatomical participants are also likely restricted. For example, the GO class 'placenta development' has an *only_in_taxon *restriction to Theria, so we propagated this to the Uberon class 'placenta'. Note that this is the reverse of the deductive inference we ought to make-we should in fact infer that 'placenta development' is only in Theria from the fact that 'placenta' is only in Theria. However, a well-populated set of GO biological processes plus taxonomic constraints existed prior to Uberon, necessitating working in this backwards direction.

### Augmentation using Allen Brain Atlas

We downloaded the OWL version of the Allen Brain Atlas (ABA) and aligned it using the methods described above. We took advantage of the fact that the ABA, like most atlases, provides a non-overlapping parcellation, and derived spatial disjointness axioms to add to Uberon.

The ABA is a partonomy that is represented in OWL as a subclass hierarchy. For every axiom in ABA of the form A DisjointWith B, we derived an axiom (*part_of *some A') DisjointWith (*part_of *some B'), where A' and B' are the Uberon equivalents of A and B. We represented this in the ontology using the *spatially_disjoint_from *shortcut relation (see below). For example, ABA contains the axiom:

ABA:HPF DisjointWith ABA:Isocortex

We used this to derive an axiom:

(*part_of *some UBERON:0002421) DisjointWith (*part_of *some UBERON:0001950)

where UBERON:0002421 has the label 'hippocampal formation' and UBERON:0001959 has the label 'neocortex'.

These axioms were used to detect problems in Uberon, some of which could be traced back to source ontologies (see, for example, [77]).

### Periodic re-alignment with external ontologies

At semi-regular intervals we re-align with existing AOs in case new terms have been added that are in scope for Uberon, or if label or synonym changes reveal new equivalencies. We also examine the change logs for different ontologies and manually check these against the ontology contents.

Since its initial inception, we have occasionally added new ontologies to the set, which we align and provide connecting axioms to. The most recent addition has been SNOMED [78]. At this time we do not formalize the connection to SNOMED and maintain the mappings as semantics-free cross-references in the obo file, as additional work will be required to determine the exact semantics of the mappings due to the Structure-Entire-Parts (SEP) construction of SNOMED.

### Manual curation of the ontology

After initial seeding of the ontology, we relied more on manual edits rather than lexical methods. We used a combination of the literature and of our own domain knowledge to expand, refine and populate the ontology. We also relied heavily on the knowledge curated in existing ontologies. We provide the source of textual definitions and in some cases individual relationships. Per-relationship provenance is stated using *trailing qualifiers *in OBO format, which translate to *axiom annotation *in OWL.

We used a combination of OBO-Edit and emacs to edit the ontology, and a combination of OBO-Edit, Protege4 and the blipkit graphviz tool to visualize and explore the ontology. We also use a collection of *ad hoc *scripts [79] for ontology processing and manipulation.

### Encoding OWL axioms in OBO format

One limitation of working in OBO format is the reduced expressivity compared with OWL. For example, OBO format cannot directly encode GCI axioms such as:

(*part_of *some 'spinal cord') DisjointFrom (part_of some 'brain')

that is, the brain and spinal cord share no parts in common. An OWL reasoner will flag any class that violates this as unsatisfiable.

Whilst we will likely switch to having the editors version of the ontology be OWL at some point in the future, we found it very convenient to remain in OBO format during initial development due to its simplicity and familiarity to contributing biologists. We made use of the 'shortcut relationship' macro feature of OBO format 1.4 [80] to encode GCIs and other advanced OWL features within OBO format.

For example, we defined a shortcut relation *spatially_disjoint_from*, which is associated with the macro:

*has_part *exactly 0 (part_of some ?Y)

This allows us to state that the brain and the spinal cord share no parts using a simple pairwise relationship.

### Maintenance, release and availability

We continuously maintain the editor's version of the ontology, which is called 'uberon_edit', and periodically make releases of the main Uberon file. The editor's version is periodically realigned with existing scAOs to harvest cross-references from newly generated classes, usually when the external ontologies change.

The release pipeline involves invoking the OBO-Edit Rule Base Reasoner to automatically build the full subsumption graph (this releases the ontology authors from the tedious and error-prone chore of maintaining the full graph). After this we perform a number of automated checks, including: synonym check-no two classes should share either labels or exact synonyms (sharing labels with weaker synonym scopes is allowed); disjointness violation check and equivalence check-if the reasoner infers two classes are equivalent then we go back and repair the ontology before releasing it.

We use the OBO Ontology Release Tool (Oort; Dietze H, in preparation) to generate release ontologies. Oort is responsible for converting the editors' version to OWL, expanding the shortcut relationships (see Materials and methods section), and generating the taxonomic bridge axioms (Additional file 1).

Uberon is housed in a github repository and is made available via the OBO registry and the http://uberon.org website. It is available as a 'minimal' ontology, with the links to other scAOs represented as cross-references, and also available as a multi-merged ontology, which has all referenced ontologies included, together with SubClass links. Uberon exists in two versions-an editor's version, with a minimal number of asserted links, and a deployed version, with equivalent links that have been pre-reasoned [81]. Additional file 1 summarizes all the available ontology versions.

## Abbreviations

ABA: Allen Brain Atlas; AO: anatomy ontology; BTO: Brenda Tissue Ontology; CARO: Common Anatomy Reference Ontology; CL: Cell Type Ontology; EFO: Experimental Factor Ontology; EHDAA/EHDAA2: Edinburgh Human Developmental Anatomy: abstract version/abstract version 2; FBbt: FlyBase Anatomy Ontology; FMA: Foundational Model of Anatomy; GCI: General Class Inclusion; GO: Gene Ontology; MA: Mouse Anatomy Ontology; MIREOT: Minimum Information Reference to an External Ontology Term; MPO (also MP): Mammalian Phenotype Ontology; NIF: Neuroscience Information Framework; OBI: Ontology of Biomedical Investigations; OBO: Open Biomedical Ontologies; OWL: Ontology Web Language; scAO: species-centric anatomical ontology; TAO: Teleost Anatomy Ontology; vHOG: vertebrate Homologous Ontology Group; XAO: *Xenopus *Anatomy Ontology; ZFA: Zebrafish Anatomy Ontology.

## Authors' contributions

CM drafted the manuscript, performed the computational aspects and assisted with manual editing of the ontology. MH co-wrote the manuscript and oversaw the biological validation of the ontology. CT contributed to the ontology imports management and Figure 3. GG assisted with the biological validation and checked the mouse-human results, and ensured consistency with phenotype ontologies. SL supervised the development of the ontology and co-wrote the manuscript. All authors have read and approved the manuscript for publication.

## Supplementary Material

Additional file 1 **Supplemental Table 1**. Table describing means of accessing the different Uberon sub-modules, available in different formats. Click here for file

Additional file 2 **Supplemental Table 2**. Table with all relations used in Uberon, together with their definitions, IDs, and usage. Click here for file

Additional file 3 **Supplemental Table 3**. Table of all classes in FMA (human) whose instances develop from the pharyngeal arches. Click here for file

Additional file 4 **Supplemental Table 4**. Table of all classes in MA (mouse) whose instances develop from the pharyngeal arches. Click here for file

Additional file 5 **Supplemental Table 5**. Table showing list of issue tracker items deriving from consistency checks performed using Uberon. Click here for file
